# Is ipsilateral administration of COVID-19 vaccine boosters the optimal approach?

**DOI:** 10.1016/j.ebiom.2023.104852

**Published:** 2023-10-31

**Authors:** Mildred A. Iro, Matthew Buckland

**Affiliations:** a Paediatric Infectious Diseases and Immunology, The Royal London Hospital, Barts Health NHS Trust, London, United Kingdom; b Department of Immunology, Royal London Hospital, Barts Health NHs Trust, London, United Kingdom; c Department of Immunology, Great Ormond Street Hospital for Children NHS Foundation Trust, London, United Kingdom

Ziegler and colleagues^1^ studied the impact of administering a second dose of the COVID-19 vaccine (BNT162b2) in the ipsilateral or contralateral side to the first dose. Although ipsilateral and contralateral vaccination induced strong humoral and cellular immune responses, secondary boosting was enhanced with ipsilateral vaccination. Additionally, ipsilateral vaccination generated higher spike-specific neutralising antibody activity and greater numbers of individuals mounting sufficient spike-specific CD8 T-cell responses.

Muecksch and colleagues^2^ found that a third COVID-19 mRNA vaccine dose was associated with an increase in, and evolution of receptor binding domain specific memory B cells from expanded memory B cell clones present after the second dose and the emergence of new clones. In animal studies, a higher number of antibody-forming cells in draining versus non-draining lymph nodes is seen following vaccination.^3^ Whilst these findings shed light on the possible immunological mechanisms that support ipsilateral vaccination and the recommendation by Ziegler and colleagues, contralateral vaccination may still be beneficial by boosting or expanding a less well utilised pool of responder memory cells.

Although the findings by Ziegler and colleagues serve as food for thought, some aspects need consideration. First, the timing of the second vaccine dose does not reflect current guidance. It is also unknown whether the findings would be replicated with a heterologous or repeated COVID-19 booster strategy. Thus, it is unknown whether the findings can be easily translated to routine use. These aspects should be carefully considered when evaluating the impact of ipsilateral versus contralateral site administration for COVID-19 vaccines.

## Declaration of interests

We declare no competing interests.
